# Supplementary material for: Causal Relationship between Aging and Anorexia Nervosa: A White-Matter-Microstructure-Mediated Mendelian Randomization Analysis
Source: Biomedicines. 2024 Aug 16;12(8):1874. doi: 10.3390/biomedicines12081874 (PMC11351342; doi:10.3390/biomedicines12081874)
Supplement: Supplementary file 1 [file biomedicines-12-01874-s001.zip › Supplementary Figure/Figure S9/Leaveoneout plot of MR analysis for ubm-b-1772 to AN.pdf]

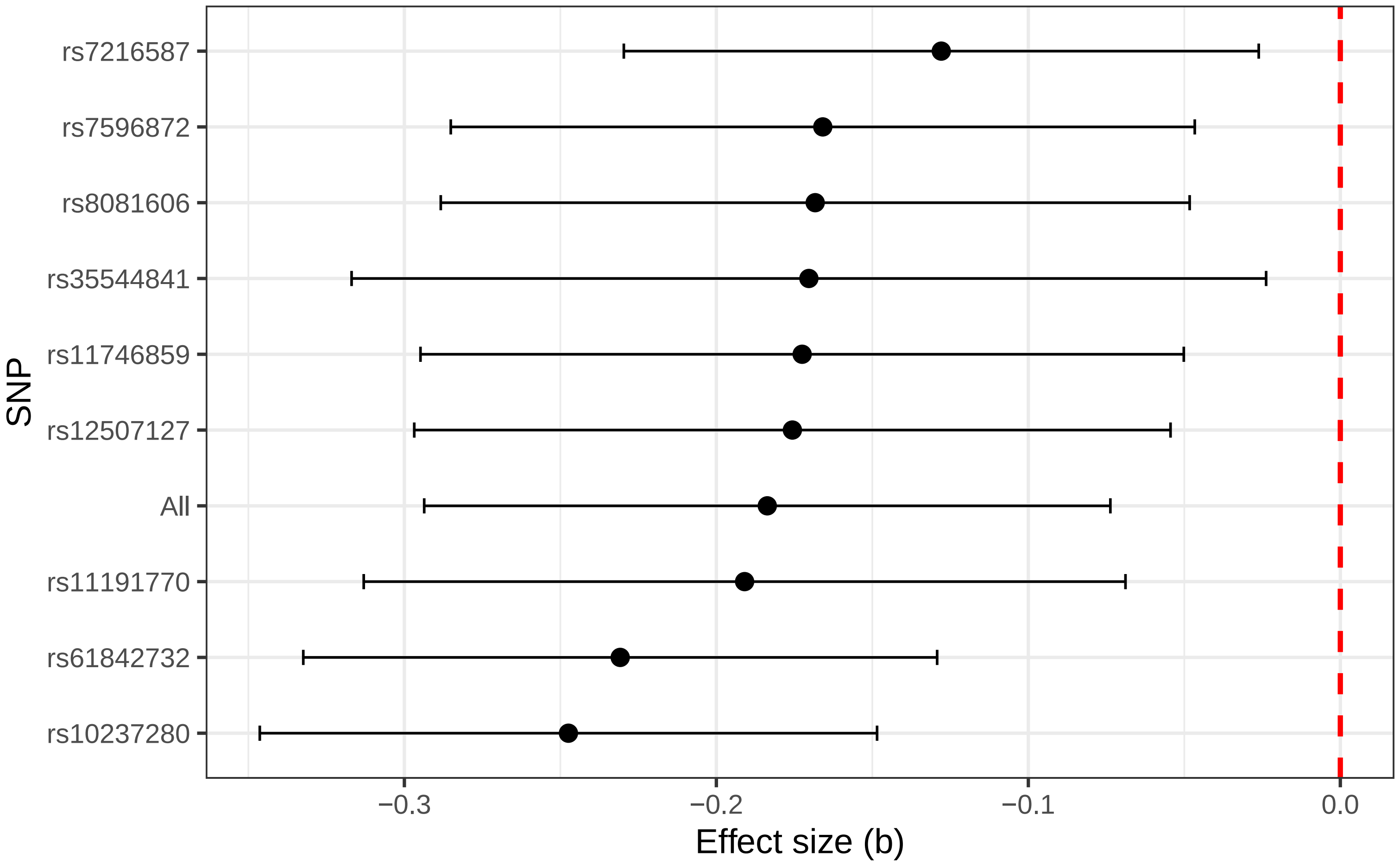
rs7216587
rs7596872
rs8081606
rs35544841
rs11746859
rs12507127
All
rs11191770
rs61842732
rs10237280
SNP
−0.3
−0.2
−0.1
0.0
Effect size (b)
